# Draft Genome Sequence of *Methanobacterium* sp. Strain 34x, Reconstructed from an Enriched Electromethanogenic Biocathode

**DOI:** 10.1128/MRA.01138-19

**Published:** 2019-11-07

**Authors:** Ala’a Ragab, Dario R. Shaw, Krishna P. Katuri, Pascal E. Saikaly

**Affiliations:** a Water Desalination and Reuse Center, Biological and Environmental Science and Engineering Division, King Abdullah University of Science and Technology (KAUST), Thuwal, Saudi Arabia; Indiana University, Bloomington

## Abstract

A draft genome sequence of *Methanobacterium* sp. strain 34x was reconstructed from the metagenome of an enriched electromethanogenic biocathode operated in a microbial electrosynthesis (MES) reactor. *Methanobacterium* sp. strain 34x has 68.98% nucleotide-level genomic similarity with the closest related methanogen available with a whole-genome assembly, Methanobacterium lacus strain AL-21. This genome will provide insight into the functional potential of methanogens at the biocathodes of MES systems.

## ANNOUNCEMENT

Electromethanogenesis is the CO_2_ reduction to CH_4_ catalyzed by hydrogenotrophic methanogens using a poised cathode as the electron donor, directly or indirectly, via H_2_ generated on the cathode surface from the hydrogen evolution reaction. The *Methanobacterium* spp. are known to dominate the biocathodes of electromethanogenic reactors (1–3). While most studies report some microbial community analyses, there is a general lack of genome-level analyses (4). Here, we report a draft genome of a methanogen, *Methanobacterium* sp. strain 34x, reconstructed from the metagenome of replicate biocathodes. The biocathodes were inoculated with anaerobic membrane bioreactor sludge and enriched for 5 months in single-chamber microbial electrolysis cells (MECs) on carbon cloth cathodes and a DSMZ 826 medium (lacking fumarate) with 10 mM acetate as a carbon source (5). They were further enriched for 6 weeks in dual-chamber microbial electrosynthesis (MES) reactors using only CO_2_ as an externally added carbon source. The enriched biocathodes catalyzed electromethanogenesis at a poised potential of −0.8 V compared to the standard hydrogen electrode (SHE).

Genomic DNA and total RNA were coextracted from the biocathodes using the PowerBiofilm RNA isolation kit (Qiagen, Germany) following a modified protocol. Approximately 0.25 g (0.2 to 1 cm^2^) of the biocathode was placed into bead-beating lysing matrix E tubes (MP Biomedicals, New Zealand) with 100 μl phenol-chloroform-isoamyl alcohol (pH 6.5 to 8.0) (Amresco, Inc., USA). Samples were physically disrupted for 2 × 40 s at 6 m/s in a FastPrep-24 (MPBio) instrument with a 3-minute resting time between cycles. After centrifugation at 13,000 × *g* for 1 minute at room temperature, the supernatant was further processed according to the manufacturer’s instructions. The extracted DNA concentration was measured using a Qubit double-stranded DNA (dsDNA) high-sensitivity (HS) assay kit (Thermo Fisher Scientific, USA) and fragmented to approximately 550 bp using an M220 ultrasonicator (Covaris, Inc., USA). DNA libraries were prepared using the NEBNext Ultra II DNA library preparation kit according to the manufacturer’s instructions (Illumina, USA). The DNA libraries were paired-end sequenced (2 × 150 bp) on the NextSeq 550 system (Illumina, USA), generating approximately 5.9 million reads. Adapter and quality trimming of the reads was performed with Cutadapt version 1.10 (6) with a minimum phred score of 20 and a minimum length of 150 bp. The trimmed reads were assembled with SPAdes version 3.7.1 (7) and mapped back to the assembly using Minimap2 version 2.5 (8) to generate the coverage files for metagenomic binning. Genome binning was carried out in R version 3.3.4 (9) using the R-studio environment. Individual genome bins were extracted using the multimetagenome principles (10) implemented in the mmgenome2 R package version 2.0.13 and refined manually as described in the mmgenome package version 0.7.1 (11). The genome bins recovered are entirely reproducible from the raw metagenome assemblies using the R files available at https://github.com/DarioRShaw/Cathode-set-potential. The majority (∼68 to 92%) of the metagenomic reads from two replicate biocathode samples were assigned to the extracted bin, which was annotated with PROKKA version 1.12-beta (12). Phylogenomic analysis was done based on 139 single-copy core genes (13) and concatenated using the Anvi’o workflow for phylogenomics (14). Default settings were used for all software unless otherwise noted.

A 2.2-Mbp genome sequence comprising 80 contigs (GC content of 37% and *N*_50_ value of 72,243 bp) was obtained, and 2,218 gene-coding regions, 48 tRNAs, and a single rRNA operon were annotated. Based on CheckM version 1.0.5 analysis, the extracted genome bin had a completeness of 98.80%, a contamination level of 9.20%, and a strain heterogeneity of 100% (15).

Phylogenomic analysis indicated that strain 34x belongs to the genus *Methanobacterium* of the family *Methanobacteriaceae*. The calculated average nucleotide identity with the closest related *Methanobacterium* sp. available with a whole-genome assembly (Methanobacterium lacus strain AL-21 [GenBank accession number CP002551]) was 68.98%, suggesting that this genome represents a novel species.

### Data availability.

The *Methanobacterium* sp. strain 34x draft genome sequence reported here has been deposited in the GenBank database under the accession number VCMF00000000. The Sequence Read Archive (SRA) accession numbers are SRR9192478 and SRR9192479. The BioProject accession number is PRJNA543631.
